# Antioxidant and ACE Inhibitory Activity of Enzymatic Hydrolysates from *Ruditapes philippinarum*

**DOI:** 10.3390/molecules23051189

**Published:** 2018-05-16

**Authors:** Yue Yu, Fengjiao Fan, Di Wu, Cuiping Yu, Zhenyu Wang, Ming Du

**Affiliations:** 1 School of Food Science and Technology, National Engineering Research Center of Seafood, Dalian Polytechnic University, Dalian 116034, China; yyindlpu@163.com (Y.Y.); m13039998695@163.com (D.W.); yuguiyan@126.com (C.Y.); wangzy0506@outlook.com (Z.W.); 2 Department of Food Science and Engineering, Harbin Institute of Technology, Harbin 150090, China; fanfjklyx@163.com

**Keywords:** *Ruditapes philippinarum*, hydrolysates, molecular weight distribution, amino acid composition, antioxidant activity, ACE inhibitory

## Abstract

Ruditapes *philippinarum* proteins were hydrolyzed by trypsin, neutrase, and pepsin. The antioxidant activities and ACE inhibitory activity of hydrolysates were analyzed and the antioxidant activities were related to their molecular weight distribution and amino acid compositions. Results indicated the hydrolysis of proteins led to an increase in small peptides and free amino acids. The antioxidant activities of *Ruditapes philippinarum* hydrolysates against DPPH radical scavenging, inhibition on linoleic acid peroxidation, and reducing power showed that the neutrase hydrolysate exhibited the strongest antioxidant activity. In addition, an ACE inhibition assay revealed that the pepsin hydrolysate had the highest ACE inhibitory ability. *Ruditapes philippinarum* protein hydrolysates could be a promising source of natural antioxidant and ACE inhibitory.

## 1. Introduction

Marine organisms constitute approximately half of the total biodiversityticals [1]. Bioactive peptides are specific fragments of protein that have plenty of potential physical functions within the body, include antioxidant, immunomodulatory, antibacterial, antithrombotic, antihypertensive activity, and so on [2]. In addition, some peptides may exhibit multifarious properties [3].

*Ruditapes philippinarum* (*R. philippinarum*), an edible species of saltwa all over the world, is a rich resource of structurally diverse biofunctional components [4]. In particular, some marine organisms are ideal raw materials for the generation of protein-derived bioactive peptides, which can be applied in food, health-care products, and pharmacy. The *R. philippinarum*, clam of the Veneridae family, is widely distributed on the coasts of China, Korea, Japan, America, and Spain [5]. This clam is commercially harvested, being the second most important bivalve grown in aquaculture worldwide [6]. The *R. philippinarum* is also a nutrient-rich food and contains minerals, vitamins, amino acids, and proteins that can be developed into value-added products by enzymatic hydrolysis. Some studies have indicated that the glycogen and taurine in *R. philippinarum* could be used to cure many chronic diseases such as arteriosclerosis and hepatitis. There are plenty of studies that have reported bioactive peptides derived from seafood sources demonstrate properties including antioxidant activity, ACE inhibitory activity, antimicrobial activity and so on. However, few studies have evaluated multifarious activities of hydrolysates of *R. philippinarum*, and the information regarding changes in the functional properties, antioxidant and ACE inhibitory activities during the hydrolysis was limited [7,8].

Reactive oxygen species (ROS) have been implicated in the etiology of biochemical and physiological lesions [9], such as cancer, cardiovascular disease, diabetes, disorders, neurodegenerative, and aging [10]. The human body has a defensive system against ROS bases on endogenous antioxidants (i.e., glutathione) and antioxidant enzymes (i.e., catalase, superoxide dismutase and glutathione peroxidase). But when ROS overloads the body’s antioxidant defenses, or when the antioxidant defense system loses its capacity for response (e.g., the aged), the oxidative stress occurs and may damage vital components of cellular [11]. So the antioxidants are demanded for they can neutralize oxidative reactions, lower the concentration of free radicals. There are many synthetic antioxidants in the market, such as butylated hydroxytoluene, butylated p-hydroxyanisole and propyl gallate, they are low-cost and effective but may cause potential risks in vivo, which limited their use in foodstuffs and have been prohibited in some countries [12]. Therefore, searching for healthy and natural antioxidants has drawn extensive attentions in recent years [13].

The antioxidant activity of protein hydrolysates could be attributed to not only a single antioxidant mechanism; hence, the antioxidant properties should be assessed by different reaction mechanisms in vitro, such as reducing power, inhibition activity on lipid peroxidation, and scavenging activity on free radicals [14]. There are some studies have indicated that some factors, such as molecular weight [15,16], hydrolysate concentration [17], degree of hydrolysis (DH) [18], and amino acid compositions [19], can affect the antioxidant activity of protein hydrolysates.

The renin angiotensin system (RAS) plays a crucial role in blood pressure and is known as the major factor in the pathophysiology of cardiovascular diseases [20]. The angiotensin I was produced by renin from angiotensinogen, then cleaved by angiotensin I-converting enzyme (ACE) to liberate a potent vasoconstrictor, angiotensin II. ACE also has a depressor action, to inactivate bradykinin. Thus, a primary target of antihypertension is the inhibition of ACE. Many synthetic ACE inhibitors (e.g., captopril) may cause side effects such as taste alterations, cough and skin rashes. Therefore, more and more researches have been conducted based on identification of foods as potential natural sources of ACE inhibitors. Many bioactive peptides with ACE inhibitory activity have been extracted from protein hydrolysates of animal sources [21,22,23].

The aim of this work was to produce hydrolysates containing potential biopeptides using various commercially available enzymes, and to evaluate the antioxidative activities and ACE inhibitory activities of the hydrolysates. The impacts of degree of hydrolysates, molecular weights and amino acid composition to the functional properties were evaluated for further applications as food additions or diet nutrients.

## 2. Results and Discussions

### 2.1. Typical Characteristics of Hydrolysates

The proximate composition on a dry weight basis of the RPHs (the *R. philippinarum* hydrolysates) was listed in Table 1. It was observed that the major composition of hydrolysates was protein (50.96–55.79%), and the protein contents of the hydrolystates have no significant difference. The results indicated that the RPHs are suitable as protein sources for developing high-valued functional products. However, there was about 14.59~19% of ash in hydrolysates, as we all know Cl^−^ and Na^+^ are the primary composition of the ash, so desalination would be necessary if the hydrolysates were used in food industry.

**Figure 1:**
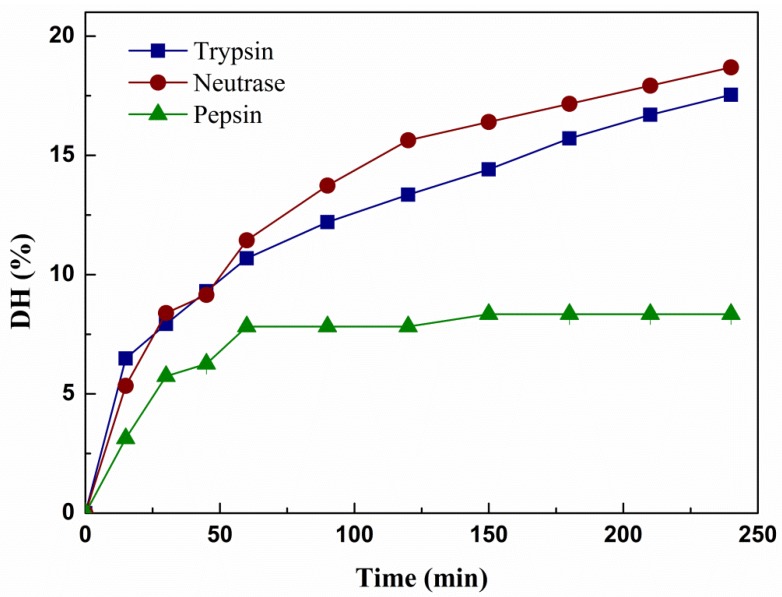
Changes of the degree of hydrolysis of *R. philippinarum* protein during hydrolysis by trypsin, neutrase and pepsin. Hydrolysis conditions: Neutrase 3.0%, 45 °C, pH 6.5; Trypsin 3.0%, 45 °C, pH 9.0. Pepsin 3.0%, 45 °C, pH 4.0.

The *R. philippinarum* hydrolysates were prepared by trypsin, neutrase and pepsin, which correspondingly produced three disparate hydrolysates, hydrolysate of *R. philippinarum* by trypsin (tRPH), hydrolysate of *R. philippinarum* by neutrase (nRPH) and hydrolysate of *R. philippinarum* by pepsin (pRPH). Figure 1 demonstrated the changes in DH of the hydrolysates during the course of hydrolysis, all hydrolysis curve increased rapidly at the first 50 min, then the hydrolysis speed of trypsin and neutrase gradually decreased, but still showed steady rate up to the end of 4 h hydrolysis. Meanwhile, the hydrolysis speed of pepsin remain nearly unchanged. The highest DH for *R. philippinarum* protein was produced with neutrase (18.30%) at 240 min reaction time, followed by trypsin (17.54%) and the DH of hydrolysate by using pepsin was only 8.34%. The rate of hydrolysis decreased could owing to reduced number of susceptible peptide bonds. It can be concluded that, the enzymatic activity of the neutrase was least affected by the resultant peptides and the substrate concentration changed [24].

### 2.2. MW Distribution of the Hydrolysates

**Figure 2:**
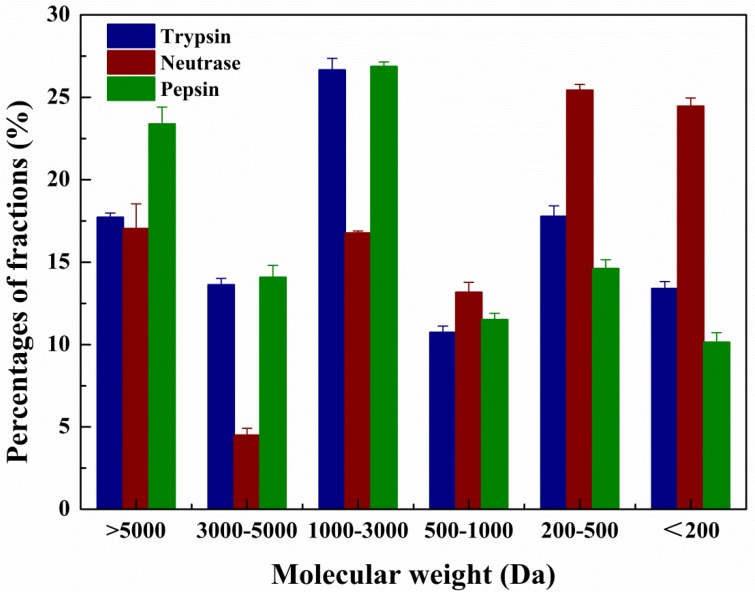
Molecular weight distribution of *R. philippinarum* protein hydrolysates. Results represent means ± SD of three independent experiments. Values with p < 0.05 were considered significant.

MW distribution is a significant parameter in the assessment of protein hydrolysates, which maybe influence their antioxidant activities and other functional properties [25]. Qin et al. has exposed that protein hydrolysates mainly consist of different molecular weight peptides [26]. The MW distribution of RPHs was determined by HPLC equipped with a Superdex Peptide 10/300 GL column. As shown in the Figure 2, during the process of hydrolysis, not all of the proteins of *R. philippinarum* were decomposed into peptides with MW below 5000 Da, and the pepsin produced the highest content of high MW fraction (>5 kDa). Hydrolysis of neutrase continued with increasing time and the high MW fractions shifted toward the lower MW fractions, and the number of low MW peptides increased. The relative proportions of low molecular-weight peptides (<1000 Da) produced by trypsin, neutrase and pepsin were 41.92%, 63.06% and 36.27% respectively. The hydrolysis activity could have resulted from specific cleavage site in substrates. Neutrase produced the highest content of low MW fraction since it is an endopeptidase which has no specific enzyme cutting site [27]. While trypsin has high cleavage specificity, it cleaves the C-terminal to arginine and lysine residues [28], and Rodriguez et al. postulated that it tends to cleave before proline [29], so the proteins were increased susceptibility to neutrase and trypsin digestion. The poor activity of pepsin could be explained by its specific cleavage site, since pepsin was clarified that had a strict preference for cleaving the peptide bonds between hydrophobic and aromatic residues and showed limited and slow peptic digestion of the proteins [30], so it failed to cleave proteins efficiently because of the limited number of aromatic amino acids [31].

### 2.3. Amino Acid Composition Analysis

The amino acid compositions of hydrolysates are shown in Table 2. All of the hydrolysates produced by three enzymes contained all essential amino acids (37.77–43.62% of the total amino acids) and were abundant in glycine, alanine, arginine, glutamic acid, and lysine, indicating that the hydrolysates of *R. philippinarum* can probably be an ideal protein supplement to unbalanced protein for nutrition improvement. nRPH has higher content and percentages of most amino acid residues compared with tRPH and pRPH, except for serine, cysteine, arginine, and asparagine. In our view, the reason why three hydrolysates have difference in amino acid compositions can be attributed to the distinction in specificity of those proteases, since the neutrase has no specific enzyme cutting site while trypsin and pepsin both have strict cleavage specificities.

Plenty of studies have verified a good correlation between antioxidant ability of protein hydrolysates and certain amino acid residues [32]. For example, Zainol et al. has demonstrated that aromatic amino acids (i.e., tyrosine and phenylalanine) could contribute to the scavenging of free radicals by acting as potent electron donors, some hydrophobic amino acids (i.e., alanine, leucine, and proline) were proved to conduce to the free radicals scavenging activity [33,34]. Peptides which have high contents of methionine, leucine, histidine, alanine, and valine, would possess strong antioxidant capacity [35,36]. Therefore, the hydrolysate produced by neutrase would be expected to exhibit optimal antioxidant ability due to the high content of these amino acid residues with better antioxidant activity.

### 2.4. Inhibitory Activity on Linoleic Acid Peroxidation

Lipid peroxidation is an oxidative chain reaction that radical-mediated abstraction of hydrogen atoms from methylene carbons in polyunsaturated fatty acids; in the emulsified linoleic acid system, alkoxyl and peroxyl were demonstrated be derived from the pre-existing lipid peroxide and were directly acted on initiate lipid peroxidation [37].

**Figure 3:**
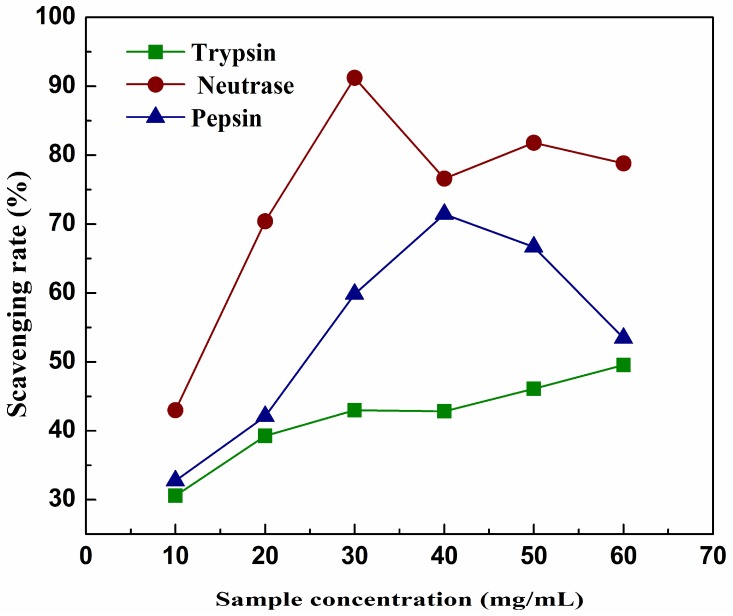
Antioxidant activities of the hydrolysates on linoleic acid emulsion in the FTC method during 4 h hydrolysis by trypsin, neutrase and pepsin with different concentrations. Results represent means ± SD of three independent experiments.

As shown in Figure 3, all of the three hydrolysates were effective inhibitors with >50% inhibition of linoleic acid oxidation when the concentration of the sample reached a higher level (IC_50_ of nRPH = 14.59, IC_50_ of tRPH = 59.62, IC_50_ of pRPH = 26.89). The fact that the hydrolysate is a mixture contains antioxidative and pro-oxidative components might result in less efficient antioxidant ability against lipid oxidation [38]. The sequence of inhibition activity was: nRPH > pRPH > tRPH. High proportion of hydrophobic amino acids may have contributed to the antioxidant activities of hydrolysates [39]. This study showed similar trends with some other studies that the lower molecular weight fraction (<1000 Da) exhibited a significant antioxidant activity [36,37]. The reason why hydrolysates of low molecular weight exhibited antioxidant activity may due to them acting as chain-breaking antioxidants by inhibiting radical-mediated peroxidation of linoleic acid [40]. The maximal values of each hydrolysate on inhibition activity were: 91.23% (nRPH), 71.56% (pRPH), and 49.56% (tRPH). The hydrolysate produced by trypsin has a dose relationship between concentration and inhibition activity, but the inhibition rate of nRPH and pRPH gradually decreased after reached a certain concentration. It may due to the decomposition and instability of hydrogen peroxides to other subordinate oxidation products after prolonged incubation [17].

### 2.5. DPPH Scavenging Activity

The DPPH radical model is widely used to investigate the free-radical-scavenging ability of various samples. DPPH is a free radical which can become a stable diamagnetic molecule when accepts a hydrogen radical or electron. In the DPPH radical-scavenging assay, the method is quick and scavenging activity is noticeable as a change in color from purple to yellow.

**Figure 4:**
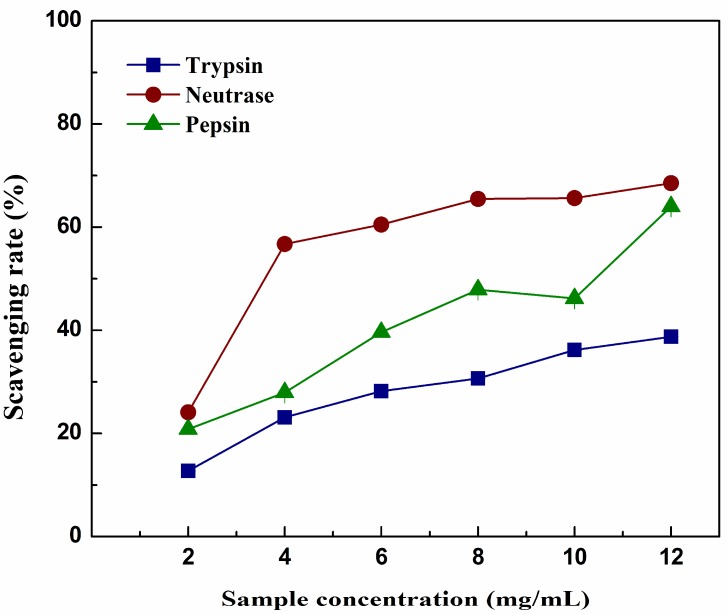
DPPH (2,2-diphenyl-2-picrylhydrazyl) free radical scavenging activities of 4 h hydrolysates during hydrolysis by trypsin, neutrase, and pepsin with different concentrations. Results represent means ± SD of three independent experiments.

A concentration-dependent assay was carried out with different hydrolysates and the results are presented in Figure 4. It was apparent that hydrolysates produced by different enzymes all exhibited significant scavenging activities on the DPPH radical; their scavenging rate raised with the increasing concentration. The antioxidant activities were in the following order: nRPH > pRPH > tRPH. The maximal values of each hydrolysate on scavenging activity were: 68.55% (nRPH), 63.98% (pRPH) and 38.77% (tRPH). The results agreed with some studies that the specificity of enzymes may influence the DPPH scavenging activity of hydrolysates. In addition, hydrolysate with low molecular weight distribution has better scavenging activity than which with high molecular weight [25].

### 2.6. Reducing Power

**Figure 5:**
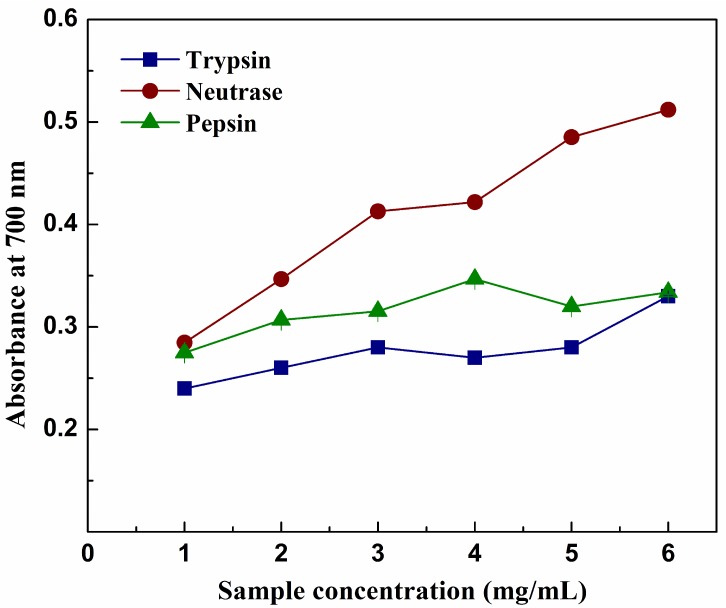
Reducing power of the different hydrolysates during 4 h hydrolysis by trypsin, neutrase and pepsin with different concentrations. Results represent means ± SD of three independent experiments.

The reducing capacity of a given sample may play a significant role as indicator of its potential antioxidant activity. It was measured by estimate transformation of the ferric iron (III) to ferricyanide complex to the ferrous iron (II). In addition, compounds with higher reducing power may have better capacities to donate hydrogen or electron [41]. The reducing power of hydrolysates is shown in Figure 5, increasing absorbance demonstrated higher reducing power potency. Hydrolysates of different concentrations exhibited a dose-dependent effect on the reducing power (absorbance at 700 nm). The nRPH had the strongest reducing power, followed by the pRPH and tRPH, which agreed with the radical scavenging activity. The maximal values of each hydrolysate of absorbance were: 0.51 (nRPH), 0.35 (pRPH), 0.33 (tRPH). The result indicated that compared with trypsin and pepsin, hydrolysate prepared by neutrase possibly had more active peptides or amino acids, which could contribute to form more stable compounds with free radicals.

### 2.7. ACE inhibitory Activity

**Figure 6:**
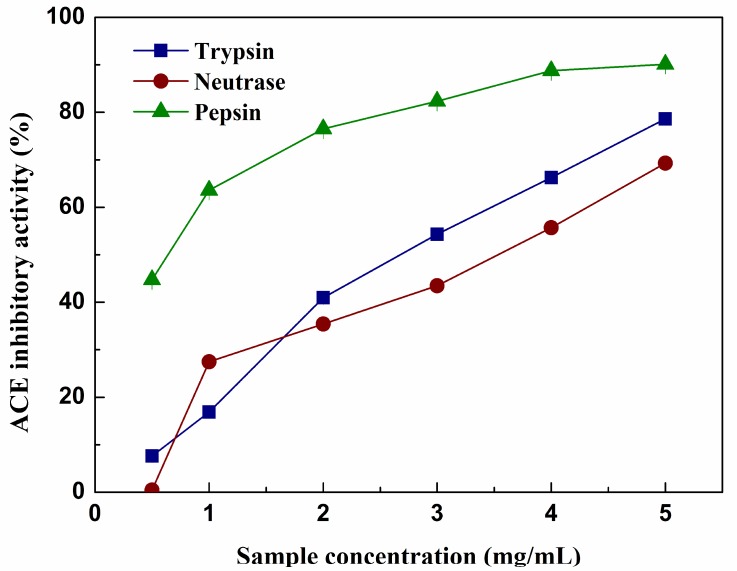
ACE inhibitory activity of the different hydrolysates during 4 h hydrolysis by trypsin, neutrase, and pepsin with different concentrations. Results represent means ± SD of three independent experiments.

ACE activity leads to an increase in the blood pressure by cleaving angiotensin I to antiotensin II and degrading the vasodilator peptide bradykinin. Thus ACE has been identified as a primary factor in hypertension, inhibitors of ACE have been widely utilized to prevent the production of angiotensin II in order to cure cardiovascular diseases [42]. There have been many studies based on foodborne peptides to exhibit ACE inhibitory activity, those peptides are deemed to be safer and milder than synthetic drugs. In addition, some peptides may be multifunctional and easy to be absorbed in human gastrointestinal tract [43]. In this sense, the ACE inhibitory activity of hydrolysates was analyzed. As shown in Figure 6, the ACE inhibitory activity exhibited by three hydrolysates suggests that the peptides released from these proteins caused this inhibition. The activity was concentration dependent, the values increased with increasing amount of samples, but didn’t increase with increase in DH. The results agreed with the work of Mullally and Lv in which they found the ACE inhibitory activity had no correlation with the DH [44,45]. The pRPH exerted the highest inhibitory activity (IC_50_ = 0.42 mg/mL) when concentration reached 5 mg/mL, while at the same concentration, the IC_50_ of tRPH and nRPH was 2.93 and 3.53 mg/mL respectively. The difference in ACE inhibitory activity may be attributed to the differences in chain length and amino acids sequences of peptides as well as to their hydrophobicity [46]. Theodore and Kristinsson reported that small peptides did not appear to increase the activity of the whole hydrolysate, but rather decreased it [47]. This is the case of the nRPH. It exhibited the lowest ACE inhibitory activity, although it had the highest amount of low MW fraction since the DH was the highest.

## 3. Materials and Methods

### 3.1. Materials

Fresh clams *R. philippinarum* (shell length of 3–4 cm, dry weight of 0.3–0.4 g) were collected from a commercial market (Dalian, China). Trypsin (EC 3.4.21.5), neutrase (EC 3.4.24.4), pepsin (EC 3.4.23.1) were acquired from Solarbio (Beijing, China). Amino acid standards, tween 20, linoleic acid, ascorbic acid, thiobarbituric acid (TBA), butylated hydroxytoluene (BHT), the 2,2-diphenyl-2-picrylhydrazyl (DPPH), potassium ferricyanide, ferric chloride, 2,2′-azinobis-(3-ethyl-benzthia-6-sulfonic acid) (ABTS), trifluoroacetic acid (TFA), hippuric acid, angiotensin converting enzyme from rabbit lung and N-Hippuryl-His-Leu hydrate (HHL) were all obtained from Sigma-Aldrich (Milwaukee, WI, USA). The other used reagents and chemicals were at analytical grade and commercially available.

### 3.2. Preparation of Enzymatic Hydrolysates from R. philippinarum

The hydrolysis conditions used in this study were summarized in Table 3. Before enzymatic hydrolysis, *R. philippinarum* was lyophilized and pulverized into powder. 2.5 g of lyophilized *R. philippinarum* powder was added to 50 mL of milli-Q water at 45 °C, after stirring for 3 min, pH was adjusted to the conditions described in Table 3, and then different enzymes were added in the mixture with enzyme to substrate ratio of 3:100 (*w*/*w*). The enzymatic hydrolysis reactions were performed for 4 h at the optimal pH adjusting by NaOH or HCl. Agitation was kept at a rate of 60 rpm. At the end of hydrolysis, the mixture was heated to 100 °C and maintained for 10 min to inactivate the enzyme. Finally, the temperature and pH of hydrolysates were all adjusted to 25 °C, 7.0 and the hydrolysates were centrifuged at 14,400× *g* for 10 min. The supernatant was collected, lyophilized and stored at −30 °C. Protein hydrolysates were clarified by filtering through 0.45 μm filters to remove the insoluble substrate and residual enzyme.

DH is defined as the percent ratio of the number of peptide bonds cleaved during the hydrolysis to the total number of peptide bonds in the substrate and can be calculated by the following Equations (1) and (2):

(1) $$DH(\%)=\frac{BN_{b}}{M_{p}\alpha h_{tot}}\times 100$$

(2) $$\alpha =\frac{\mathrm{10}pH-pK}{1+10pH-pK},\;pK=\frac{7.8+2400\times (298-t)}{298(273.15+t)}$$

In this formula, *B* is the amount of NaOH or HCl added to the substrate to keep the pH constant during the hydrolysis; *N_b_* is the normality of NaOH or HCl, it is 0.1 M in the present study; α is the degree of dissociation of the α-NH2 groups liberating during hydrolysis; *M_p_* is the mass of protein (in grams), which was 1.25 g in this experiment; *h_tot_* is the total number of the peptide bonds which is assumed to be 7.5 meq.g; and *pK* is the dissociation value (7.5) for the α-amino groups releasing during hydrolysis, it is 7.5 in this study.

### 3.3. Proximate Compositions Analysis

The moisture, ash, protein and lipid contents of the *RPHs* were determined according to the methods of AOAC (2000). Moisture content was measured by using a drying oven at 105 °C. Total ash content was determined in a muffle furnace by heating the sample at 550 °C for 8–12 h. Total nitrogen content of *RPHs* was measured by using Kjeldahl method (Foss Kjeltec Nitrogen Analyzer, Model 8400, Stockholm, Sweden), content of protein was obtained by multiplying the factor of 6.25 by total nitrogen value. Crude fat content was estimated by extracting with diethyl ether for about 6h using soxhlet apparatus.

### 3.4. Molecular Weight Distribution

The molecular weight (MW) distribution of *RPH*s was determined by gel-permeation chromatography, by using an Elite P230 HPLC system (Elite Analytical Instruments Co., Ltd., Dalian, China) equipped with a Superdex Peptide 10/300 GL column (300 × 10 mm, GE Healthcare Co., Little Chalfont, Buchinghamshire, UK) according to the method of Wu et al. with some modifications [23]. 10 µL of RPH solution (4 mg/mL, filtered through 0.45 μm filter) were loaded onto the HPLC with mobile phase: acetonitrile/water/trifluoroacetic acid = 30:70:0.1 (*v*/*v*/*v*) at a flow rate of 0.4 mL/min and monitored at 220 nm by an ultraviolet (UV) detector. The cytochrome c (12,500 Da), aprotinin (6512 Da), vitamin B12 (1355 Da), GSH (307 Da) and glycine (75 Da) were used as the MW standards. The standards yielded a linear retention time (x) versus log MW (y) regression curve in the MW range of 75–12,500 Da: *y* = −0.0835*x* + 5.9666 (R^2^ = 0.9955). The chromatogram was grouped into six parts according to the retention time: >5000, 5000–3000, 3000–1000, 1000–500, 500–200, and <200 Da, provided the profilogram of different molecular weight compositions. The percentage areas under the curve stand for the percentage content of peptide fraction.

### 3.5. Determination of Free Amino Acid Composition of RPHs

We used a targeted HPLC–MS/MS method for determination of 20 free amino acids composition with some modification [48]. Hydrolysates were pretreated by a one-step protein precipitant extraction for analysis: 0.1 g lyophilized powder of hydrolysates was completely dissolved in water (with 0.1% formic acid and 2% methyl alcohol, *v*/*v*), then 1 volume of sample solution 4 volumes of acidified acetone (with 0.4% HCl, *v*/*v*) were mixed and kept at −20 °C overnight, to allow protein precipitated adequately, the mixture was then centrifuged at 14,400× *g* and supernatant was extracted and lyophilized. Analysis was performed on a high-performance liquid chromatography (HPLC, Shimadzu LC-10AD binary pump, Kyoto, Japan) coupled with a triple quadrupole mass spectrometer (AB Sciex, 4000Qtrap System, Framingham, MA, USA). Separation was conducted with an AB Sciex AAA C18 column (4.6 × 150 mm, 4.5 µm particle size) at a flow rate of 0.8 mL/min. Mobile phase A comprised 0.1% formic acid in Milli-Q water. Mobile phase B comprised 0.1% formic acid in acetonitrile. The lyophilized powder was dissolved in 1 mL of acidified water (0.1% formic acid, *v*/*v*), the injection volume was 1 µL using a gradient elution system: 0–0.5 min, 0–2% B; 0.5–10 min, 2–25% B; 10–10.1 min, 25–90% B; 10.1–12 min, 90% B; 12–12.1 min, 90–2% B; 12.1–18 min, 2% B. Positive mode and multiple reaction monitoring (MRM) was selected to acquire the sample data. Qualitative and quantitate of each amino acid in hydrolysates were determined by the retention time and chromatographic peak areas of amino acid standard (data not shown). Data analysis was performed on Agilent Quantitative Analysis version B.06.00 analyst data processing software (Agilent Corporation, Beijing, China).

### 3.6. Inhibitory Activity on Linoleic Acid Peroxidation

To assess the in vitro lipid peroxidation inhibition activity of hydrolysates, a thiobarbituric acid (TBA) method was carried out, which reported by Kapila et al. with slight modification [49]. 200 µL of linoleic acid was emulsified with 400 µL of Tween 20 in 19.4 mL of PBS (0.02 M, pH 7.4), then the mixture was sonicated for 10 min to gain a homogeneous emulsion. 500 µL of linoleic acid emulsion was mixed with 600 µL PBS (0.02 M, pH 7.4), 0.2 mL of ascorbate (0.01%), 200 µL of FeSO_4_ (0.01%), and 500 µL of each hydrolysate, with concentrations ranging from 10–60 mg/mL and incubated at 37 °C. After incubation for 24 h, the 2 mL reaction solution was mixed with 200 µL of TCA (4%), 200 µL of butylated hydroxyl toluene (BHT, 0.4%) and 2 mL of TBA (0.8%), the mixture was incubated at 100 °C for 30 min then cooled and centrifuged, supernatants were measured at 534 nm by a microplate reader, used Milli-Q water replace the sample as blank. All experiments were conducted in triplicate. The inhibition rate of linoleic acid peroxidation was defined as:

(3) $$\mathrm{Linoleic}\;\mathrm{acid}\;\mathrm{peroxidation}\;\mathrm{inhibition}\;\mathrm{activity}(\%)=\frac{A_{\mathrm{BLANK}}-A_{\mathrm{SAMPLE}}}{A_{\mathrm{BLANK}}}\times 100$$

where *A*_BLANK_ stands for the absorbance of control reaction and *A*_SAMPLE_ is the absorbance of the hydrolysates.

### 3.7. DPPH Scavenging Activity

The scavenging activity on the 1,1-diphenyl-2-picrylhydrazyl (DPPH) free radical of the hydrolysates was measured according to the method of Suda et al. with slight modifications [50]. The reaction solution contained 200 µL of each hydrolysate, 400 µL of phosphate buffer (0.1 mol/L, pH 6.0) and 400 µL of 0.2 mmol/L DPPH in 95% ethanol. After shaking, the mixture was immediately moved to dark and stand for 30 min, the absorbance of the mixture was recorded at 517 nm by a microplate reader. The percentage of scavenging effect was calculated as:

(4) $$\mathrm{DPPH}\;\mathrm{scavenging}\;\mathrm{rate}(\%)=\frac{1-(A_{1}-A_{0})}{A_{2}}\times 100$$

where *A*_1_ was the absorbance of each hydrolysate, *A*_0_ was the absorbance of the control (95% ethanol replaced DPPH), and *A*_2_ was the absorbance without DPPH.

### 3.8. Reducing Power

The reducing power of the hydrolysates was measured by using the method described by Chen et al. [17]. The reaction solution containing 1.0 mL of each hydrolysate, 1.0 mL of phosphate buffer (0.2 mol/L, pH 6.6) and 2 mL of potassium ferricyanide (1%, *w*/*v*) were incubated at 50 °C for 20 min. 1.0 mL of trichloroacetic acid solution (10%, *w*/*v*) was added to terminate the reaction, then the mixture solution was centrifuged at 3040× *g* for 20 min. The supernatant of solution (1 mL) was mixed with 1.25 mL of distilled water and 0.15 mL of ferric chloride (0.1%, *w*/*v*), then the absorbance of the mixture was measured at 700 nm by a microplate reader. The absorbance of the reaction mixture represented the reducing power of hydrolysates.

### 3.9. Angiotensin I-Converting Enzyme (ACE) Inhibition Assay

The ACE inhibitory activity of hydrolysates was determined following the method described by Cushman et al. with some modifications [51]. 20 µL of samples were mixed with 50 µL of 5 mmol/L HHL (in 0.1 M sodium borate buffer containing 0.3 M NaCl, at pH 8.3), and the mixture was preincubated at 37 °C for 5 min, then the reaction was started by adding 20 µL of 0.1 U/L ACE, mixture was incubated at 37 °C for 60 min. Finally, the mixture was terminated by adding 10 µL of 0.2 M HCl, used the same volume of distilled water instead of sample as the control. The separation was performed by an Agilent+1260 HPLC (Agilent Technologies, Beijing, China) with a Ghall 12S05-2546 C18 column (250 × 4.6 mm, 5 mm, Eka Chemicals AB Sciex, Framingham, MA, USA) of 0.5 mL/min and detected at 228 nm. 10 µL of reaction solution was filtered through 0.45 µm cellulose filters and loaded into the HPLC to quantify the hippuric acid produced by the enzymatic hydrolysis of the substrate hippuryl-l-histidyl-l-leucine. Mobile phase A comprised 0.05% trifluoroacetic acid in Milli-Q water, mobile phase B was acetonitrile. The elution gradient was as follow: 1–14 min, 20% B; 14–19 min, 5% B; 19–24 min, 95% B, 24–30, 20% B. The OpenLAB CDS software (version: C.01.07.SR2) was employed for data processing. The inhibitory activity was calculated by the following formula:

(5) $$\mathrm{ACE}\;\mathrm{inhibitory}\;\mathrm{activity}(\%)=\frac{A_{\mathrm{BLANK}}-A_{\mathrm{SAMPLE}}}{A_{\mathrm{SAMPLE}}}\times 100$$

where *A*_BLANK_ represented the peak area of reaction blank; *A*_SAMPLE_ was the peak area of the reaction mixture.

### 3.10. Statistical Analysis

All experiments were carried out at least in triplicate. The data were reported as mean ± standard deviation. Statistical calculations were performed by using the SPSS software (version 16.0; SPSS Inc., Chicago, IL, USA).

## 4. Conclusions

In this study, *R. philippinarum* was enzymatic hydrolyzed by several commercially available enzymes, and some properties of hydrolysates were analyzed. DH and protease greatly influenced the molecular weight and free amino acid compositions of hydrolysates. The antioxidant activities of hydrolysates were related to DH and the enzyme used. Hydrolysate produced by neutrase exhibited the strongest antioxidant activity (including inhibition activity on linoleic acid peroxidation, DPPH radical scavenging activity, and the reducing power). Furthermore, the hydrolysates also showed potential antihypertensive activity measured by an ACE inhibition assay. However, further identification of bioactive peptides in the hydrolysates and in vivo experiment were necessary if the hydrolysates being further utilized.

## Figures and Tables

**Table 1:** Proximate composition of hydrolysates from *R. philippinarum* proteins.

Composition	Trypsin	Neutrase	Pepsin
Moisture (%)	11.95	14.64	14.64
Protein (%)	55.79	53.60	50.96
Fat (%)	2.01	2.10	3.50
Ash (%)	18.13	14.59	19.00

**Table 2:** Free amino acid compositions of hydrolysates from *R. philippinarum* proteins.

Amino Acid	Trypsin		Neutrase		Pepsin	
	mg/Kg	%	mg/Kg	%	mg/Kg	%
Glycine	139.62	15.56	186.96	11.52	106.73	14.43
Alanine	94.00	10.48	195.67	12.06	80.25	10.85
Serine	22.49	2.51	29.05	1.79	8.98	1.21
Threonine	27.28	3.04	67.05	4.13	16.17	2.19
Aspartic acid	13.43	1.50	26.19	1.61	18.69	2.53
Glutamic acid	76.81	8.56	138.31	8.53	90.57	12.24
Cysteine	1.80	0.20	2.32	0.14	1.84	0.25
Proline	14.68	1.64	39.07	2.41	29.25	3.95
Lysine	88.87	9.91	113.60	7.00	33.73	4.56
Methionine	9.61	1.07	26.79	1.65	10.78	1.46
Arginine	165.16	18.41	168.36	10.38	89.31	12.07
Valine	29.30	3.27	99.22	6.12	32.51	4.39
Glutamine	22.15	2.47	72.62	4.48	19.13	2.59
Asparagine	1.65	0.18	5.02	0.31	0.73	0.10
Histidine	4.79	0.53	24.29	1.50	4.10	0.55
Phenylalanine	35.51	3.96	64.04	3.95	38.65	5.22
Tryptophan	13.32	1.48	27.10	1.67	13.02	1.76
Tyrosine	37.66	4.20	59.57	3.67	31.62	4.27
Leucine	62.41	6.96	182.86	11.27	82.07	11.09
Isoleucine	36.71	4.09	94.29	5.81	31.68	4.28
Total hydrophobic amino acids	295.54	32.94	729.03	44.94	318.22	43.01
Total essential amino acids	331.07	36.90	707.71	43.62	279.47	37.77
Total	897.26	100.00	1622.38	100.00	739.83	100.00

**Table 3:** Conditions summary of enzymatic hydrolysis.

Enzyme	Optimum Conditions		Time (h)	E:S	DH (%)
	pH	Temperature (°C)			
Trypsin	9.0	45	4	3/100	17.54%
Neutrase	6.5	45	4	3/100	18.30%
Pepsin	4.0	45	4	3/100	8.34%
